# Monitoring results of wild boar (*Sus scrofa*) in The Netherlands: analyses of serological results and the first identification of *Brucella suis* biovar 2

**DOI:** 10.1080/20008686.2020.1794668

**Published:** 2020-10-26

**Authors:** Peter van Tulden, Jose L. Gonzales, Michiel Kroese, Marc Engelsma, Frido de Zwart, Dorota Szot, Yvette Bisselink, Marga van Setten, Miriam Koene, Peter Willemsen, Hendrik-Jan Roest, Joke van der Giessen

**Affiliations:** a Wageningen Bioveterinary Research (WBVR), Lelystad, The Netherlands; b National Institute of Public Health and the Environment (RIVM), Bilthoven, The Netherlands

**Keywords:** *B. suis* biovar 2, MLVA- 16, PCR, multi-species *Brucella* ELISA, The Netherlands, wild boar

## Abstract

In Europe, wild boar populations pose an increasing risk for livestock and humans due to the transmission of animal and zoonotic infectious diseases, such as African swine fever and brucellosis. *Brucella suis* is widespread among wild boar in many European countries. In The Netherlands the prevalence of *B. suis* among wild boar has not been investigated so far, despite the high number of pig farms and the growing wild boar population. The Netherlands has a *Brucella*-free status for the livestock species. The objective of this study is to investigate the presence and distribution of *B. suis* in wild boars in The Netherlands and to assess the value of the different laboratory tests available for testing wild boars. A total of 2057 sera and 180 tonsils of wild boar were collected between 2010 and 2015. The sera were tested for *Brucella* antibodies and the tonsils were tested for *Brucella* spp. *B. suis* biovar 2 was detected by MLVA/MLST and culture in wild boar from the province of Limburg, while seropositive wild boar were obtained from the provinces of Limburg, Noord Brabant and Gelderland suggesting the northwards spread of *B. suis* biovar 2. In this paper, we describe the first isolation of *B. suis* biovar 2 in wild boar in The Netherlands.

## Introduction

Porcine brucellosis, caused by *Brucella suis*, is a chronic disease characterized by sterility and abortion in sows, mortality in piglets, and orchitis in (wild) boars [1]. It occurs in many countries and can cause important economic losses in the pig industry. Brucellosis in livestock is therefore a notifiable disease in many countries including The Netherlands. *B. suis* is a small gram-negative bacterium and comprises five different biovars with different host specificities. *B. suis* biovars 1,2 and 3 are maintained in domestic pigs (*Sus scrofa domesticus*) and wild boar (*Sus scrofa*). *B. suis* biovar 4 is maintained in reindeer (*Rangifer tarandus*). *B. suis* biovar 5 is isolated from rodents [2]. *B. suis* biovars 1, 3 and 4 can cause severe disease in humans, while *B. suis* biovar 2 has rarely been found in humans, mainly causing chronic disease symptoms [3]. The geographical distribution of *B. suis* biovar 2 has been described throughout continental Europe in a broad geographic area between Scandinavia, southern European countries and the Balkans [2], and is present in wild boars and European hare (*Lepus europaeus*). These wildlife species have been implicated as the source of transmission of *B. suis* biovar 2 to (outdoor reared) domestic pigs and occasionally to cattle [4,5]. On the contrary spillover of *B. suis* biovars 1 and 3 from wildlife species to domestic pigs is rare [2,6,7]. Although wild boars are predominantly known as carriers and reservoirs for *B. suis* biovar 2, the disease is seldom seen in wild boar. Seroprevalence in Eurasian wild boar of *B. suis* biovar 2 range from 8% to 32% throughout continental Europe [8] but can be as high as 55% locally [9].

In The Netherlands, the last cases of porcine brucellosis (*B. suis* biovar 1) in pigs were reported in 1969. They were related to swill feeding of imported hares from South America. Since then porcine brucellosis has not been reported in The Netherlands [10]. Little is known about the current presence and, if present, distribution of *B. suis* in wild boar populations in The Netherlands, although wild boar populations in The Netherlands are rapidly increasing due to influx from adjacent countries and natural population growth [11]. This may pose a risk for commercially kept pigs predominantly when these are free ranging.

In The Netherlands, over 11 million domestic pigs are kept at approximately 4000 farms of which approximately 2750 to be known as specialized pig farms. The percentage of farms with free-range pigs is unknown (Central Buro Statistics, 2018). The highest domestic pig density is found in the eastern and southeastern provinces of The Netherlands: Gelderland, Brabant and Limburg. Since the Dutch pig density is very high and farms are often closely located to each other, the Dutch pig farming industry is vulnerable for large outbreaks of infectious diseases like (African or Classical) Swine Fever (ASF or CSF) and Foot and Mouth Disease (FMD) [12]. Economic losses can be significant, as was shown during the last CSF outbreak in The Netherlands in 1997/1998 with an estimated € 2 billion in costs [13]. Knowledge of the pathogens circulating in wildlife reservoirs, including wild boar populations, is required to prevent the risk of disease transmission to domestic pigs.

A wild boar is a protected species in The Netherlands under the Dutch nature conservation law. Wild boars were extinct by 1826 due to intensive hunting but were re-introduced in 1904 as hunting animals [14] by the Dutch Royal family. Wild boar are permitted in so called designated areas, located in two provinces: Gelderland (Veluwe region and National Park ‘De Hoge Veluwe’ in the Central Eastern part of The Netherlands) and Limburg (National Park ‘De Meinweg’ adjacent to the border with Germany, in the Southeastern part of The Netherlands). Outside these designated areas a zero-tolerance policy is maintained, meaning no wild boars are allowed outside these designated areas. However, it was recently shown that wild boars have a much wider distribution [15,16] and the wild boar population size depends strongly on the availability of food. Nowadays, the population exceed the permitted numbers and varies roughly between 3000 and 10,000 wild boars, depending on food availability, mildly winters and hunting activities and the time of year.

A monitoring program for wild boar in The Netherlands has been established since 1994 for the early detection of notifiable diseases such as Classical Swine fever (CSF) [17]. *Brucella* spp. was and is not included and is considered as a low risk disease for the Dutch livestock farming industry. Publications from Belgium [5,9] Germany [18] and France [3] however, indicate that *B. suis* in wild boars is present in countries neighboring The Netherlands.

Therefore, the objective of this study was to investigate the presence and distribution of *B. suis* in wild boar in The Netherlands and to assess the value of the different laboratory tests available.

## Material and methods

### Sample collection

For this study blood and tonsils, using convenience sampling, were collected from an ongoing national wild boar monitoring program and were used for analyses. Depending on the population size, between 300–600 blood samples were taken by hunters every year from 2010 until halfway 2015 as part of the national monitoring program during the period of this study which stopped halfway 2015. Most wild boars were shot by registered hunters within the annual hunting season, for wild boar, (62%) between 1 July and 31 January. Also outside the wild boar hunting season, a substantial number of wild boars were legally shot (38%), based on the zero-tolerance policy outside the designated areas. We received serum samples from designated and non-designated areas. Tonsils, from non-designated areas, were received mainly from the province of Limburg following the national monitoring program for CSF. The ‘permitted’ number of wild boars per region is roughly 1200 in the Veluwe region [15], 50 in the National Park ‘De Hoge Veluwe’ and 60 in the National Park ‘De Meinweg’. Hunters were instructed to collect approximately 10 ml of blood per wild boar after shooting. All samples were sent to the Animal Health Service (AHS) in Deventer (coordinator of the national monitoring program during this study period). From a subset of animals in the far Southern part (province Limburg) of The Netherlands tonsils were also collected, starting in December 2012. In 2014, tonsils and blood from hunted wild boars in the Veluwe region were also collected for this study. These tonsils were sent directly to Wageningen Bioveterinary Research (WBVR).

### Detection of Brucella spp. from wild boar tonsils

#### Culture

Isolation of *Brucella* spp. from tonsils was performed according to the OIE protocol [19–21]. Briefly, tissue samples of approximately 2 by 2 cm were cut into small pieces and macerated with 20 ml of beef broth using a ‘Stomacher’ machine. The mixture was inoculated onto solid (1 drop) and liquid (1 ml) Castañeda’s selective medium. Both solid and liquid media were incubated at 37ºC in 10% CO_2_. Twice, with one-week intervals, liquid cultures were re-cultured on solid Castañeda’s selective media and incubated under the same conditions. All plates were inspected weekly for 3 weeks, and this procedure was repeated for 3 weeks when samples were positive after direct testing with real-time PCR. Suspicious colonies were screened for *Brucella* spp. by slide agglutination tests using *Brucella* agglutinating sera (*B. abortus* and *B. melitensis*) (Remel Europe Ltd., UK), and its susceptibility to lysis by *Brucella* specific Weybridge (Wb) and Tbilisi (Tb) bacteriophages. With each culture batch one positive control, *B. suis* biovar 1 (Intern control nr. 1330), was included. All laboratory work with potentially *Brucella*-contaminated samples was performed within a BSL3-facility.

#### Real-time PCR

DNA was extracted from the tonsils using a DNA tissue kit (DNeasy Blood and Tissue Kit; QIAGEN, Hilden, Germany), according to the manufacturer’s instructions. In addition, DNA was extracted from suspected colonies after culturing by suspending the colony in 200 μL nuclease-free water (Sigma-Aldrich, MO, USA) and boiling at 100°C for 8 minutes, followed by centrifuging for 2 minutes at 20,000 x g. The clarified supernatant was used for real-time PCR and MLVA typing as described before [19,20].

DNA isolated from all tissue samples and all colonies was tested with a real-time PCR targeting the IS711 sequences of *Brucella* spp. [22]. A synthetic internal positive control (IPC) was added to detect possible inhibition of the reaction. The 20 μL PCR mixture contained 1 μL of the DNA extract, 1 x TaqMan Universal MasterMix (Applied Biosystems, CA, USA), 1 mM (each) of IS711 primers, 0,2 mM (each) of IPC probes, 1 U of uracil DNA glycosylase (UDG), 1 μL of IPC template DNA and nuclease-free water (Sigma-Aldrich, MO, USA). The performance of the PCR was monitored using a blank (nuclease-free water; Sigma-Aldrich, MO, USA) and a positive control (*B. abortus* ref. 544, biotype 1) at different concentrations. Amplification was carried out using a 7500 Fast real-time PCR System (Applied Biosystems, CA, USA) under the following standard conditions: an initial UDG incubation step at 37°C for 5 minutes, denaturation step at 95°C for 20 seconds and 50 cycles with two steps of 95°C for 3 sec and 60°C for 30 sec. Results were analyzed with 7500 System SDS Software version 1.4 (Applied Biosystems, CA, USA). Tissue samples and colonies were considered positive after real-time PCR (RT-PCR) if the results presented a CT value ≤36 (with sigmoid curve), inconclusive if 36> CT value <40 (with doubtful sigmoid curve) and negative if CT value ≥40, or no CT at all.

#### MLVA and MLST genotyping

To differentiate isolates into *Brucella* species and biovars, MLVA-16 clustering was performed using a selection of 16 different repeat loci markers [23]. Briefly, PCR amplification was performed using a GeneAmp PCR System 9700 thermocycler (Applied Biosystems, Foster City, California, USA) in a total volume of 25 µL containing 1x reaction buffer (Thermo Fisher), 0.1 U/µL TrueStart Taq DNA polymerase (Thermo Fisher), 2 mM MgCl_2_ (Thermo Fisher), 0.4 mM of each nucleotide (dATP, dCTP, dGTP, dUTP; Thermo Fisher), 1 μM of each primer (Eurogentec S.A., Liège, Belgium), 0.1 U/µL UDG (New England Biolabs, Ipswich, Massachusetts, USA), 1 µL template, and nuclease-free water (Sigma–Aldrich). An initial UDG incubation for 5 minutes at 37°C and denaturation/activation for 2 minutes at 96°C was followed by 40 cycles of denaturation for 30 seconds at 96°C, annealing for 30 seconds at 60°C, elongation for 30 seconds at 72°C, and finalized by an extension step of 5 minutes at 72°C. PCR products with different fluorescent dyes were diluted depending on the PCR efficiency, and pooled. From these pooled PCR products, 2 µL was mixed with 15 µL of Hi-Di formamide (Applied Biosystems) and 0.5 µL of GeneScan 600 LIZ Size Standard (Applied Biosystems). Samples were denaturated for 5 minutes at 98°C and separated on a 3130 Genetic Analyzer (Applied Biosystems). Fragment sizes were determined using Peak Scanner version 1.0 software (Applied Biosystems). The number of repeats for each locus was determined from published data [24]. Further MLVA-16 clustering was carried out as described previously [24] using Bionumerics version 6.3 (bioMérieux, Marcy l’Etoile, France).

The following reference strains were used during typing of isolated cultures by real-time PCR using MLVA method: reference strains *B. melitensis* biovar 1 (NCTC10094; 16 M), *B. abortus* biovar 1 (NCTC10093) and *B. suis* biovar 1 (NCTC10316).

For MLST analysis [19], fragmented libraries were constructed using Nextera DNA sample preparation kit (Illumina, San Diego, California, USA). Next-generation whole genome sequencing was performed by paired-end sequencing using the Illumina technology on the MiSeq instrument (Illumina). De novo assembly of the quality filtered reads was performed using ABySS-pe version 1.3.3. [25]. Bowtie2 version 0.2 (Johns Hopkins University, Baltimore, Maryland, USA) aligning was used for curation of the contigs quality by Tablet version 14.04.10 [26]. Additionally MLST typing was performed in silico with a set of MLST specific primers [27] and the assembled contigs as input. Two reference strains were used during MLST analysis: *B. melitensis* biovar 1 (NCTC 10094) and *B. suis* biovar 2 (NCTC 10510).

#### Serological analyses

Serum samples were tested for *Brucella* spp. antibodies using the ID Vet ID-Screen® Brucellosis Serum indirect-ELISA Multi-Species (ID Vet, Louis Pasteur, Grabels, France) according to the manufacturer’s recommendations. According to the manufacturer the kit can be used for domestic animals and wild animals like deer and wild boar.

Briefly, serum samples and controls (10 µl + 190 µl dilution buffer) were added to the microwells diluted at 1/20 and incubated for 45 minutes at room temperature. Plates were washed 3 times with wash solution. A multi-species IgG horseradish peroxidase (HRP) conjugate, diluted 1/10 with dilution buffer, was added to the wells. Incubation was for 30 minutes at room temperature. The wells were emptied and washed 3 times with wash solution. After washing to eliminate the excess conjugate, the substrate solution (TMB) was added. Incubation was for 15 minutes at room temperature. The resulting coloration depends on the quantity of specific antibodies presented in the specimen to be tested. The microplate was read at 450 nm with a spectrophotometer. For each sample, the S/P percentage was calculated. According to the manufacturer S/P% ≤ 110 is considered negative, a S/P% 110 < S/P% > 120 is considered doubtful and a S/P% ≥ 120 is considered indicative positive in the manufacturer multi-species approach.

### Data analyses

#### Assessment of test performance

To establish the serological cut-off value specific for wild boar, available paired tissue and serum samples (n = 180) were tested for Brucella DNA (PCR) and antibody detection (ELISA). These data were used to assess the diagnostic performance of the ELISA test, using PCR as the reference test. For this evaluation, PCR CT values ≤36 were considered positive, while higher CT values ˃36 or absence of amplification (no CT value) were considered negative (see Table 2). This PCR classification was decided following a graphical assessment where a wide overlap in S/P values between PCR negative and inconclusive results was observed (Supplementary Information, Figure S1). Receiver Operating Characteristic curves (ROC) were used to identify the diagnostic cut-off for discrimination of positive and negative sera samples. The identified cut-off was that which maximized both the Sensitivity (Se) and Specificity (Sp) of the ELISA test. Performance parameters were also assessed using the ELISA kit recommended cut-off for domestic pigs.

**Table 1.** PCR and culture results of 180 tonsil samples collected in the surveillance period 2012–2015

Result	PCR^a^	Culture
Positive	19	7
Negative	146	173
Inconclusive	15	0
Total	180	180

^a^RT-PCR CT values ≤36 were classified as positive, CT values >36 and <40 were classified as inconclusive, and CT values ≥ 40 or no CT at all were classified as negative.

**Table 2.** Performance of the ELISA test, relative to PCR

	PCR^a^ +	PCR -		Total	
**Diagnostic outcomes**					
ELISA +	16	47		63	
ELISA -	3	114		117	
Total	19	161		180	
**Performance estimates (95% Confidence Intervals)**					
Cut-off S/P value			165		
Area Under Curve			0.80 (0.73, 0.87)		
Sensitivity			0.84 (0.60, 0.97)		
Specificity			0.71 (0.63, 0.78)		

^a^PCR results with CT values > 36 were considered negative, because the specificity was better than results with CT values >40 were considered negative.

#### Seroprevalence of Brucella spp

Once the diagnostic (for wild boar in The Netherlands) cut-off had been determined, it was used to classify ELISA results as positive or negative and quantify the seroprevalence of *Brucella* spp. in The Netherlands for each year of the wild boar monitoring program. Confidence intervals for the seroprevalence were estimated using the Wilson exact method.

Changes in seroprevalence during the monitoring years and differences in seroprevalence at different age, sex or geographical location of hunted boars were assessed by fitting a multivariable logistic regression model, where the serological results (positive/negative) were the response variable, and monitoring year, age, sex, and the geographical location of the hunted wild boar (X,Y coordinates) were the explanatory variables. Potential interactions between explanatory variables were also assessed.

Data analysis was performed using the statistical software R version 3.1.3 [28]. Assessment of test performance and estimation of prevalence was done using the packages epiR and ROCR (R-Foundation: https://www.r-project.org/).

## Results

### Tested wild boar population

**Figure 1.**
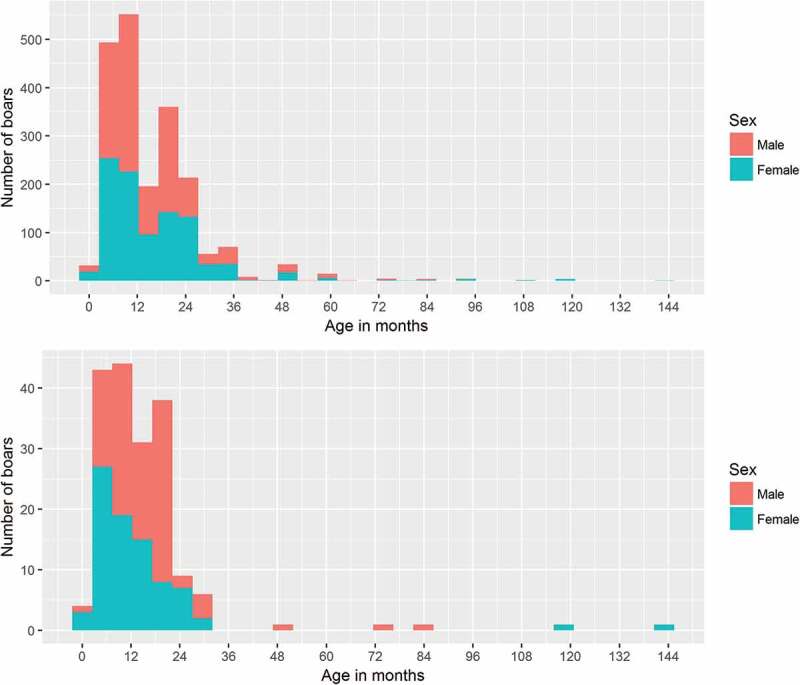
Age distribution of sampled hunted wild boars. Upper graph (a) shows the age distribution from all boars from which a serum sample was tested. Lower graph (b) shows the age distribution from all boars from which a tonsil sample was tested

Between 2010 and 2015, a total of 2057 serum samples were received for testing, which originated from the provinces of Noord Brabant, Limburg, Gelderland and Overijssel. Out of these samples, 1073 (52%) were from male and 984 (48%) from female wild boars. Age of the hunted wild boars ranged from 1 month to 144 months old, with the median age for both males and females around 12 months (Figure 1).

In addition to serum samples, a total of 180 tonsil samples were received between 2012 and 2015 originating from the province of Limburg (131 tonsils) and in 2014 also from Gelderland (49 tonsils). Out of the 180 tonsils, 97 (54%) samples were from males and 83 (46%) samples from females. The median age (range) of hunted wild boars from which tonsils were tested was 12 (1–144) months (Figure 1(b)). This is comparable with the median age of the wild boar from which serum samples were tested. Geographically, proportionally older animals were sampled in the Northern regions of the country than the Southern regions. This spatial-age distribution was similar for both the samples used (tonsils and sera) for assessment of the test and the serum samples tested to asses prevalence (data not shown).

### Detection and identification of Brucella by culture and real time-PCR

**Figure 2.**
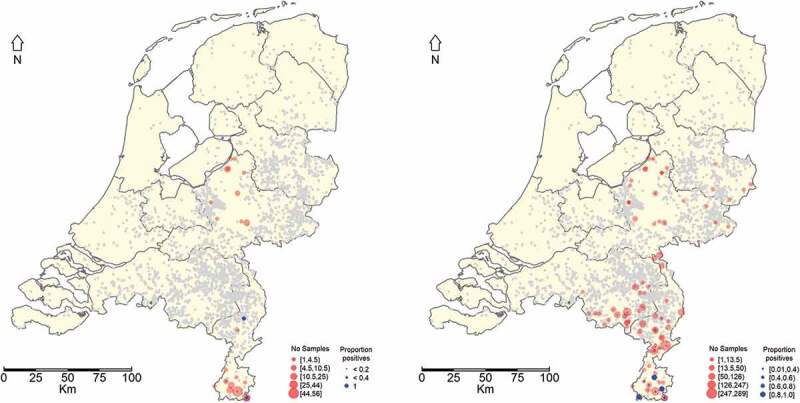
Geographical distribution of hunted wild boars from which tonsil samples were taken and observed RT-PCR results (blue dots) expressed as fraction positives (left panel). Geographical distribution of hunted wild boars from which serum samples were taken and observed ELISA test results (blue dots) expressed as fraction positives (Right panel). If no blue dot is observed then no positive result was obtained. The grey dots represent Dutch pig farms

A total of 19 samples out of the 180 were positive with PCR. Seven out of these 19 PCR-positive tonsils were *Brucella* confirmed by culture (Table 1). No *Brucella* spp. were isolated from any of the samples considered ‘Inconclusive’ by PCR (Table 1). All *Brucella* culture and PCR-positive samples originated from the southernmost province of the Netherlands, the province of Limburg (Figure 2).

### Identification of Brucella

**Figure 3.**
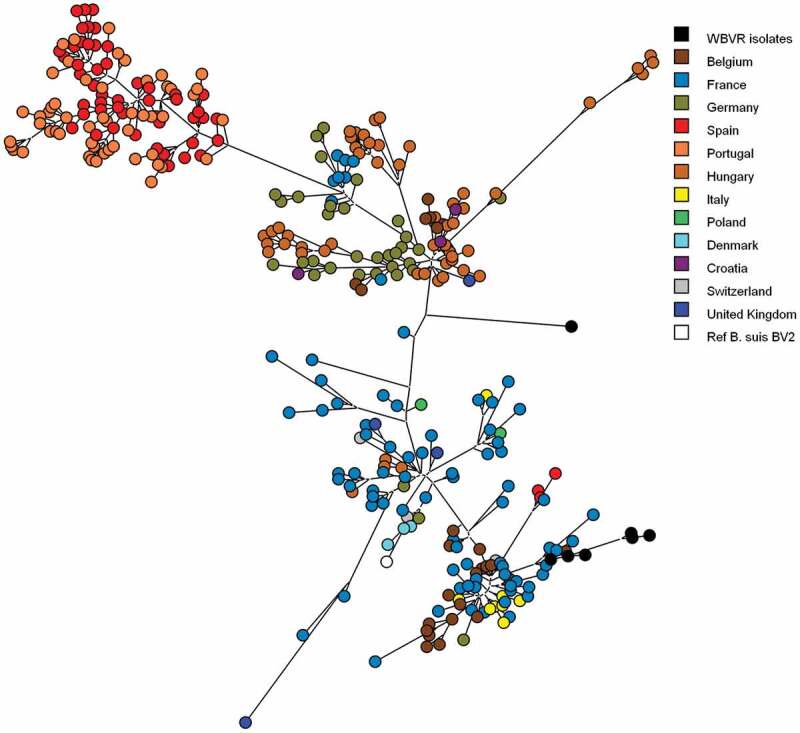
Genotypic clustering of *B. suis* biovar 2 isolates from wild boar, boar, domestic pigs, hare and reference strains using parsimony analysis of MLVA-16 clustering determined genotypes. Six WBVR isolates are clustered with strains originating from Belgium and France. One isolate could not be clustered with an geographic genotype, because the MLVA pattern was incomplete. Reference strains of *B. suis* biovar 2 was added to confirm the relationship of the isolates to be biovar 2 strains

All *Brucella* isolates from the tonsil samples were characterized as *B. suis* biovar 2 by MLVA-16 typing according to the publicly available MLVA database for *Brucella* [29], although from three isolates only an incomplete MLVA pattern could be obtained. These results were confirmed by MLST analysis showing for all seven *Brucella* isolates Sequence type 15. Using parsimony analysis on the isolated MLVA profiles combined with known *B. suis* biovar 2 isolates [23], the strains isolated from the Dutch wild boar are clustered among the *B. suis* biovar 2 strains originated from Belgium and France and 1 isolate with an incomplete pattern could not be placed in a geographic cluster (Figure 3).

### Assessment of ELISA test performance

Using PCR as a reference test, an adapted cut-off S/P value equal to 165 was identified for discrimination between positive or negative serological results situated for wild boars in The Netherlands. Table 2 summarizes the test results and the estimated diagnostic performance parameters for the ELISA assay using this cut-off value. Test performance using this cut-off was compared with that using the ELISA kit recommended multi-species cut-off (120). No significant differences (p > 0.05) in Se or Sp were observed between the two cut-off values assessed. The estimated Se and Sp at a cut-off value of 120 were Se = 0.95 (95% CI: 0.74, 1.00) and Sp = 0.66 (95% CI: 0.58, 0.73). Based on this study, we chose 165 as a species-specific cut-off to give more weight to the Sp (0.71 (95% CI: 0.63, 0.78)) of the test and therefore minimize false positive results (Table 2).

### Seroprevalence and geographical distribution

The results using the total number of serum samples tested during the study are presented in Table 3. Most samples originated from the province of Limburg, which is located in the south of The Netherlands and where the highest fraction of seropositives was found (Figure 2). The estimated seroprevalence (apparent prevalence) (Table 2) for each year of monitoring shows that the highest seroprevalence was observed in 2010 (11,6%). In this year, however, limited sampling was done and all samples originated from the province of Limburg. In the period 2011 to 2015 the geographical coverage of the sampling was higher and a yearly increase in prevalence could be observed from 4.1 to 9.6% (Table 3).

**Table 3.** Serological results of wild boar tested for *Brucella* antibodies in different provinces in The Netherlands. Results are shown as percentage positive (total number of samples)

Province	2010*	2011	2012	2013	2014	2015*	Total
Limburg	10 (86)	13 (211)	16 (280)	15 (135)	33 (295)	28 (207)	115 (1214)
% Positive	11.6%	6.2%	5.7%	11.1%	11.1%	13.5%	9.4%
Noord Brabant		1 (69)	2 (79)	1 (71)	4 (227)	2 (93)	10 (539)
% Positive		1.4%	2.5%	1.4%	1.8%	2.2%	1.9%
Overijssel				0 (1)	0 (4)	0 (7)	0 (12)
% Positive				0%	0%	0%	0%
Gelderland		0 (60)	3 (90)	0 (43)	3 (93)	0 (6)	6 (292)
% Positive		0%	3,3%	0%	3,2%	0%	2,1%
Total	10 (86)	14 (340)	21 (449)	16 (250)	40 (619)	30 (313)	131 (2057)
Adjusted prevalence % (95% CI)	11.6 (0.6,20.3)	4.1 (2.63,6.8)	4.7 (2.9,7.1	6.4 (3.7,10.2)	6.5 (4.7,8.7)	9.6 (6.6,13.4)	6.4 (5.7, 8.0)

*In 2010 and 2015 a complete year of samples could not be used or were not available.

### Risk factors

**Figure 4.**
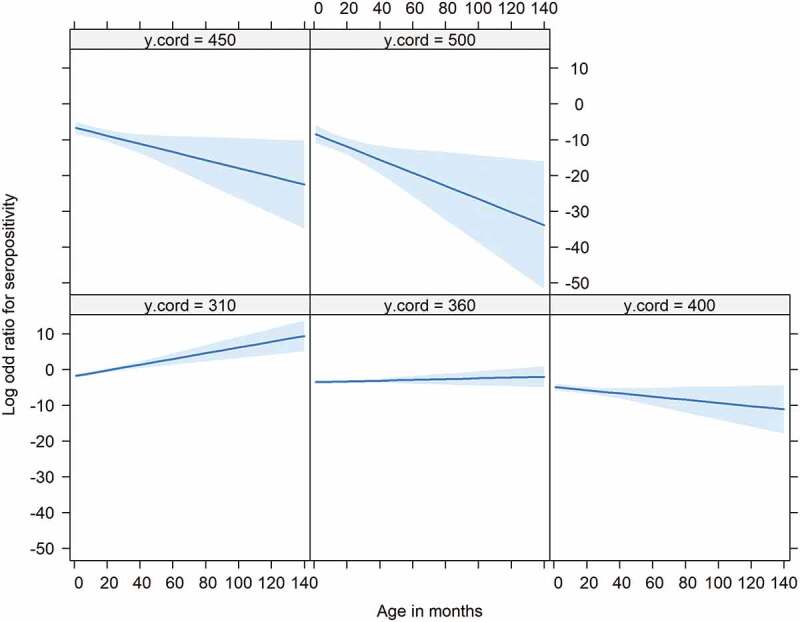
Relationship between age (X axis), odds for seropositivity (Y axis) and south-north geographic location (Facets). Facets show Y coordinates (‘y.cord’) stratified by 50 Km intervals. The higher the Y coordinate the norther the location. There is a positive increase in the odds for seropositivity with age (the older the animal the higher the odds for serpositivity) in the southern part (y coordinates ≤ 360 Km) of The Netherlands. This relationship changes in the northern regions (Y coordinates >360) with the odds for seropositivity decreasing with age (odds for seropositive higher in younger animals than older animals)

As serum samples in 2010 originated from the province of Limburg only, we excluded this year for the statistical analysis. The multivariable analysis confirmed, as observed in Figure 4, a significant decrease in prevalence along the South-North axis (Limburg – Noord-Brabant – Gelderland – Overijssel) (negative log odds for the Y coordinates (Table 4)) with the lowest prevalence expected in the northern regions of The Netherlands. The analysis also showed a significant relationship between prevalence and age. This relationship was dependent on the geographical location along the South-North axes (significant interaction between age and Y coordinate) (Table 4), with the odds of seropositivity increasing with age – the older the animal the higher the prevalence – in the Southern regions (Limburg – Noord-Brabant) whilst these odds decreased with age in the Northern regions (Gelderland – Overijssel) (Figure 4). The model also showed a significant yearly increase in prevalence between 2011 to 2015. No significant differences in seroprevalence were observed between male and female wild boars. Additionally, except age and geographical location, no significant interactions were observed between all of the other variables assessed.

**Table 4.** Effect of age, year and geographical location on the seroprevalence of *Brucella* spp. in wild boar in The Netherlands

Variable	log Odd	Odds	LCL	UCL	P
Age (months)	0.51	1.665	1.237	2.287	0.001
X coordinate	−0.003	0.997	0.974	1.021	0.799
Y coordinate	−0.034	0.967	0.952	0.982	<0.001
Year	0.213	1.237	1.068	1.438	0.005
Age: Y coordinate	−0.001	0.999	0.998	0.999	0.002

log Odd: natural log of the odds ratio, Odds: odds ratio, LCL: 95% lower confidence limit, UCL: 95% upper confidence limit and P: p-value

## Discussion

In this study, we assessed the *B. suis* presence in wild boar in The Netherlands. We tested wild boar serum using ELISA, and tissue samples of wild boar using culture and PCR assays. Nineteen tonsils tested PCR-positive for *Brucella* spp. of which 7 were also positive by culture. We found more than twice as much positives by DNA detection compared to culture, indicating that culturing is less sensitive possibly due to unculturable dead bacteria in these tissues or due to a lower detection level. It is less likely that other *Brucella* species not culturable were the cause of the PCR positives, because The Netherlands are free for *Brucella abortus, B. melitensis* and *B. suis* bv 1 in livestock and also never been detected in wildlife. Hinić et al. [30], also reported more than twice as much PCR positives compared with culture using the same IS711 marker as in our study but they used different tissues (spleen, lung and reproductive organs). Since only tonsils were available for this study, this could have influenced the number of positives detected by PCR and culture. However, these were the only tissues available for culture and PCR in the national surveillance programme. All *Brucella* isolates tested by additional typing were *B. suis* biovar 2. These results show for the first time that *B. suis* biovar 2 is present in wild boars living in the most southern part of The Netherlands adjacent to Belgium and Germany. In Belgium [5,9] and Germany [18] *B. suis* biovar 2 was reported before. As the wild boar populations in The Netherlands, Belgium and Germany are partly connected [31] to each other, it is not surprising that Dutch wild boars also tested positive for *B. suis* biovar 2. This finding is important, however, due to the economic importance of the Dutch pig industry. The introduction of *B. suis* biovar 2 in the pig population could cause serious economic losses, although a spillover from wild boar to domestic pigs has not been reported so far. Spillover of *Brucella suis* biovar 2 to cattle has, however, been reported [5] so this should also be considered when brucellosis is detected in cattle in The Netherlands.

To better assess the seroprevalence of *Brucella* spp. in wild boars in the Dutch situation, we adapted the ELISA cut-off. The commercial ELISA kit was a multispecies test with an average cut-off value and not a species-specific cut-off. Therefore, we decided to evaluate the performance of the ELISA by using the results of the PCR as a reference, since PCR appeared to be more sensitive in detecting *Brucella* spp. compared to culture. These calculations for the ELISA cut-off value were specific for the situation of wild boar in The Netherlands. Based on the best possible specificity and sensitivity given by the samples used in this study, the cut-off was adapted to 165 instead of the cut-off value provided by the manufacturer of 120. Although this decision increases the chance of false negative results, it does minimize the number of false positive results and improves therefore the positive predictive value of the test. It should be noted that we did not use a ‘perfect test’ for the evaluation of ELISA test. The fact that only tonsil samples were available and tested by PCR could have limited the Se of the PCR, with infected boars classified as negative because the bacteria was not present in tonsils but could be present in other tissues, therefore the Sp of the ELISA test is likely to be higher than our current estimates. Nevertheless, our choice of the cut-off reduced false positive results. The selection of this cut-off had no influence in the statistical analysis for the identification of risk factors. We also performed this analysis using a cut-off = 120 and the same risk factors, as those reported in Table 4, were identified as significant with similar estimated Odd Ratios (see supplementary information (Table S1). For future research, application of Bayesian methods for test validations could improve the estimates of diagnostic performance of the ELISA and the PCR tests, however some information on the Se of the PCR as a function of the sample type tested (e.g. tonsils) would be desirable to be able to use informed prior information.

The overall seroprevalence increased significantly over the years 2011 to 2015 from 4.1% to 9.6%. The seroprevalences are still much lower than those found in Belgium (up to 55%) [9] and in Germany (up to 26%) [18]. Also, the low seroprevalences compared to the surrounding countries might indicate that *B. suis* was introduced more recently in The Netherlands. Hinić et al. [30], also reported the detection of *Brucella* spp. in blood samples using PCR. For this, whole blood is necessary which was not available in this study. This analysis could give more information about the introduction of *B. suis* biovar 2. The multivariable analyses confirmed the increasing exposure of *B. suis* in wild boars between 2011 and 2015 and a significant decrease in seroprevalence along the axis South to North (longitude). The interaction between longitude and age showed an increase in seroprevalence as function of age in the Southern provinces whist the behavior was different in the Northern provinces. The seroprevalence was likely to be higher in younger than older wild boars. Although the seroprevalence is lower in the Northern regions, the appearance of seropositive cases particularly in young animals in these regions is a cause for concern. This might indicate that *Brucella* spp. is spreading northwards, facilitated by the steady increase in total numbers and geographic distribution of wild boar in The Netherlands in recent years. This increase in numbers of wild boar might be a result of the high survival rate of offspring, the availability of food, the difficulty in culling wild boar and the spread of the population [11].

This Northerly spread is also of concern due to the risk of transmission to livestock, as a larger part of the livestock population will be exposed. The highest fraction of serological and culture/PCR positives were found in the most Southern province (Limburg), where there is a low pig farm density. Most of the pig farms are located in the upper part of the province of Limburg and the provinces of Gelderland and Noord Brabant (Figure 2). These three provinces contain 80% of all the domestic pigs. This may result in a low risk of transmission from wild boar to domestic pigs in Limburg. However, in the northern region of Limburg and the above adjacent province of Noord Brabant where the domestic pig density is much higher then, given the apparent northwards spread of *Brucella* spp., the risk of transmission to domestic pigs is a cause for concern, especially where domestic pigs have outdoor access. However, the sample size analyzed in this study is limited compared to the number of culled wild boar, and the available tonsil and serum samples of wild boar were not originally intended for *Brucella* spp. A specific sampling strategy was not realized, because tonsils were only available from wild boar originating from the designated area with the highest risk for CSF, the province of Limburg.

Based on the MLVA profiles, most of the *B. suis* isolates clustered with isolates originated from Belgium and France, suggesting an influx of the infection from a Southwestern direction. Although recently, distinct and geographically coherent wild boar clusters have been identified in The Netherlands and Western Germany [31]. It is conceivable that this clustering also strongly determines the spatial distribution of *B. suis* strains in wild boar hosts. Kreizinger et al. [32], mentioned the geographic distribution of *Brucella suis* biovar 2 in countries neighboring The Netherlands might suggest an influx from Southwestern direction could be caused by hares as host species instead of the more dominant host species wild boar in Germany. Further genetic investigations are necessary to determine the dominant host species introducing *B. suis* biovar 2 in The Netherlands

## Conclusion

*B. suis* (biovar 2) is present in wild boars in The Netherlands. We isolated and identified *B. suis* biovar 2 in the southernmost part of The Netherlands and based on the gradient in the serological results, a tendency of spreading towards the Northern regions could be identified. Based on the molecular results, we hypothesize that *B. suis* entered The Netherlands from the South.

Since there is a risk of spread of *Brucella* spp. in a growing wild boar population, we recommend including *Brucella* spp. as part of the existing wild boar long-term monitoring program in The Netherlands because domestic pigs and cattle might be at risk.

## Supplementary Material

Supplemental Material
