# Supplementary material for: Monitoring results of wild boar (Sus scrofa) in The Netherlands: analyses of serological results and the first identification of Brucella suis biovar 2
Source: Infect Ecol Epidemiol. 2020 Oct 26;10(1):1794668. doi: 10.1080/20008686.2020.1794668 (PMC7595143; doi:10.1080/20008686.2020.1794668)
Supplement: Supplemental Material [file ZIEE_A_1794668_SM6510.docx]

**Supplementary Information.**


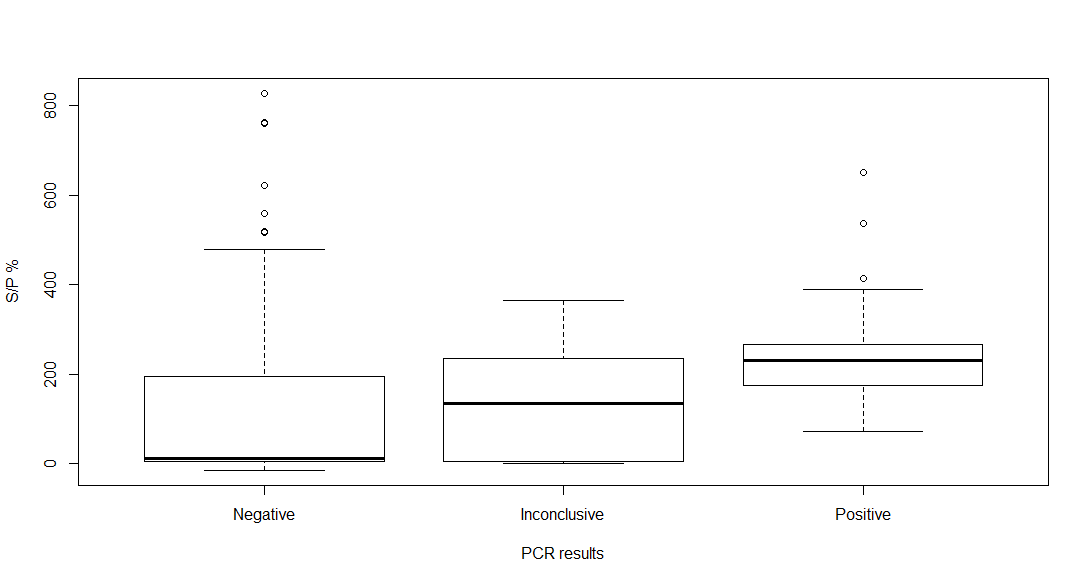


Figure S1. Distribution of the ELISA S/P values according to an initial classification of PCR CT values as: No Ct value = Negative, 36 – 40 = Inconclusive, <36 = Positive.

Table S1. Effect of age, year and geographical location on the seroprevalence of *Brucella* spp. in wild boar in The Netherlands. This analysis is done using a Cut-off value for the ELISA test = 120.

| Variable^*^ | log Odd | Odds | LCL | UCL | P |
| --- | --- | --- | --- | --- | --- |
| Age (months) | 0.565 | 1.76 | 1.326 | 2.381 | 0.000 |
| X coordinate | -0.02 | 0.98 | 0.961 | 1.001 | 0.054 |
| Y coordinate | -0.033 | 0.967 | 0.953 | 0.981 | 0.000 |
| Year | 0.219 | 1.245 | 1.086 | 1.433 | 0.002 |
| Age : Y coordinate | -0.002 | 0.998 | 0.998 | 0.999 | 0.000 |

^*^ log Odd: natural log of the odds ratio, Odds: odds ratio, LCL: 95% lower confidence limit, UCL: 95% upper confidence limit and P: p-value. Gender was not a significant risk factor.
